# Radiological Risk to Human and Non-Human Biota Due to Radioactivity in Coastal Sand and Marine Sediments, Gulf of Oman

**DOI:** 10.3390/life11060549

**Published:** 2021-06-11

**Authors:** Ibrahim I. Suliman, Khalid Alsafi

**Affiliations:** 1 Department of Physics, College of Science, Imam Mohammad Ibn Saud Islamic University (IMSIU), Riyadh 11642, Saudi Arabia; 2 Department of Radiology, Medical Physics Unit, King Abdulaziz University, P.O. Box 80215, Jeddah 21589, Saudi Arabia; kalsafi@kau.edu.sa

**Keywords:** radioactivity, gamma spectrometry, absorbed dose rates, radiation risk, aquatic environment, non-human biota

## Abstract

Natural and ^137^Cs radioactivity in coastal marine sediment samples was measured using gamma spectrometry. Samples were collected at 16 locations from four beaches along the coastal area of Muscat City, Gulf of Oman. Radioactivity in beach sand was used to estimate the radiological risk parameters to humans, whereas the radioactivity in marine sediments was used to assess the radiological risk parameters to non-human biota, using the ERICA Tool. The average radioactivity concentrations (Bqkg^−1^) of ^226^Ra, ^232^Th, ^40^K, ^210^Pb and ^137^Cs in sediments (sand) were as follows: 16.2 (16.3), 34.5(27.8), 54.7 (45.6), 46.8 (44.9) and 0.08 (0.10), respectively. In sand samples, the estimated average indoor ($D_{\mathrm{in}}$) and outdoor ($D_{\mathrm{out}}$) air absorbed dose rates due to natural radioactivity were 49.26 and 27.4 and the total effective dose (${\mathrm{AED}}_{\mathrm{Total}}$; µSvy^−1^) ranged from 150.2 to 498.9 (average: 275.2). The measured radioactivity resulted in an excess lifetime cancer risk (ELCR) in the range of 58–203 (average: 111) in and an average gonadal dose (AGD; µGy.y^−1^) ranged from 97.3 to 329.5 (average: 181.1). Total dose rate per marine organism ranged from 0.035 µGy h^−1^ (in zooplankton) to 0.564 µGy h^−1^ (in phytoplankton). The results showed marine sediments as an important source of radiation exposure to biota in the aquatic environment. Regular monitoring of radioactivity levels is vital for radiation risk confinement. The results provide an important radiological risk profile parameter to which future radioactivity levels in marine environments can be compared.

## 1. Introduction

Radioactivity naturally exists in the environment in different conditions such as soil, underground water, marine sediment, and biota. Radioactivity enters the marine environment through different pathways, including via river and rainwater transport into the sea; however, this is often due to nuclear waste disposal, which is discharged from nuclear power plants as well as from medical, industrial, research, and educational uses of radionuclides [1,2,3]. Sources of marine radioactivity are numerous: uranium isotopes are present in large amounts in seas and oceans; thorium in water is hydrolysed and attached to particle surfaces, and is thus not as soluble in water; and ^226^Ra and ^40^K are highly soluble in water. On the other hand, ^210^Pb enters the atmosphere via ^222^Rn diffusion and in rainfall. ^137^Cs in the environment poses radiation protection concerns given its high yield, long half-life, and significant uptake and retention in biological organisms. The principal sources of ^137^Cs released in the environment have included atmospheric nuclear weapons testing and releases during nuclear reactor accidents [4].

Regarding the radiation risk to non-human biota, the concerning biological effects include those that could lead to changes in population size or structure. Among these endpoints are early mortality, some forms of morbidity, impairment of reproductive capacity, and the induction of chromosomal damage. Therefore, it is necessary to estimate the doses received and then compare such data with the nearest relevant data for reference organisms to evaluate the likely radiation effects for such organisms in an environmental context [5].

Assessing radiation exposure among humans requires a better understanding of the radionuclide’s behaviour in pertinent environments [6,7,8]. Thus, the primary aims of nearly all marine radioactivity studies have been to form a scientific foundation upon which to determine the radiological risk of radioisotopes in marine environments. This is an enormously important issue that is in alignment with the present radiation protection standards [6,7]. Considering the importance of the subject, several radioactivity studies were performed in the marine environments of Gulf countries [9,10,11,12,13]. The results revealed a high degree of variability in radioactivity levels and emphasised the importance of such studies from a radiation protection standpoint. In Oman, not much work has been done to explore environmental and marine radioactivity. In fact, the only study we found was carried out by Salih, who studies radioactivity levels in marine environments [14]. In these studies, the radiological risk to non-human biota was not covered.

Thus, we sought to assess radiation exposure, radiological hazards, and attributed cancer risk from naturally occurring radioactivity found in marine sediments along the coastal area of the Gulf of Oman. This is important, as oceans and seas are directly impacted and ultimately serve as a sink of radioactivity and other contaminants, as they link diverse geographical areas with one another, representing a major source of marine pollution.

## 2. Materials and Methods

### 2.1. Study Location

**Figure 1:**
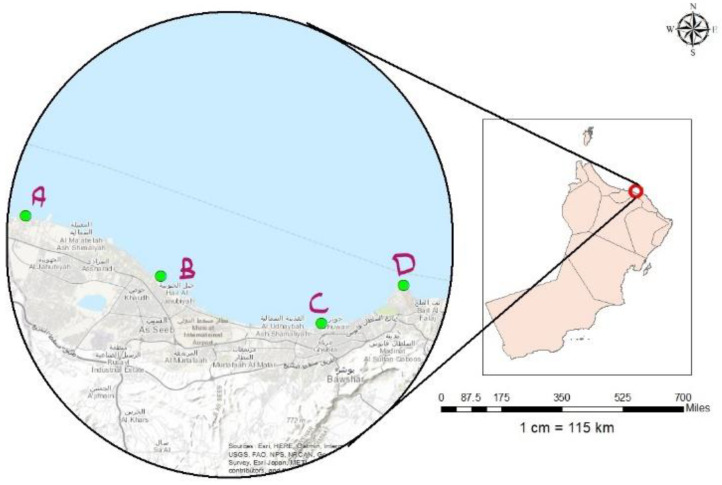
Map of Oman showing Muscat city, Gulf of Oman and the four sample locations: Manuma Beach (**A**); Seeb Beach (**B**); Aziba Beach (**C**); and Qurum Beach (**D**).

The study was performed in the Muscat principality in Oman (23.5859° N, 58.4059° E) which had a population of approximately 1.28 million in 2015. Figure 1 shows the map of Oman, which highlights Muscat city and the four sample locations. These are Manuma Beach (A), Seeb Beach (B), Aziba Beach (C), and Qurum Beach (D), which are the four major beaches. These beaches are important sightseeing destinations and are among the most abundantly frequented in the city, especially in summer. Thus, it is essential that radioactivity levels are studied to determine the extent of the associated radiological hazards.

For the coastal sands, each sample was taken at a depth of 5 cm at an average interval of 200 m between two locations. Samples of marine sediment were taken from the area covered by sea water at about 20 m from the beach to provide a fair representation of the area’s geological and sediment characteristics, which are the greatest determinants of the types of radionuclides present. The samples were taken to the laboratory at the Medical Physics Department, Sultan Qaboos University, where they were dried for 24 h in an oven set at 80 °C. To better estimate the specific activity of radium, samples were tightly sealed in Marinelli beakers and left for 4 weeks to achieve equilibrium.

### 2.2. Radioactivity Measurements

Spectrometric measurements were performed using a p-type high purity germanium (HPGe) detector, with a relative efficiency of 40% to that of NaI spectrometry (ORTEC, Oak Ridge, TN, USA). Gamma Vision-32 software was used for spectrum analysis (ORTEC, Oak Ridge, TN, USA). Energy and efficiency calibrations were performed before the measurements were taken using a standard mixture of sources from the International Atomic Energy Agency (IAEA). Background measurements were performed without a sample in place, and these measurements were subsequently subtracted from the measured activity concentrations.

The ^226^Ra activity was estimated from the ^214^Pb and ^214^Bi radionuclide activities measured directly determined from their gamma-ray energy lines 351.92 keV and 609.31 keV, respectively. The activity of ^232^Th was estimated from the ^212^Bi, ^212^Pb, and ^228^Ac radionuclide activities measured directly from their gamma-ray energy lines 727.17, 238.63, and 911.60 keV, respectively. The activity concentrations of ^40^K, ^210^Pb, and ^137^Cs were measured directly using their gamma ray lines 1460.81, 46.5, and 662 keV, respectively [4]. Using these parameters, the specific activity (A) of a given radionuclide in the sample was determined as follows:

(1) $$A=\frac{N}{\mathrm{PE}\cdot ℰ\cdot \mathrm{Tc}\cdot M\cdot k}$$

where M is the mass of the sample in kg, N is the sample net area in the peak range, PE is the gamma emission probability, Tc is the counting time, and ℰ is the photo peak efficiency [7]. *k* is the product of all correction factors ($k=k_{1}\cdot k_{2}\cdot k_{3}\cdot k_{4}\cdot k_{5}$); where $k_{1}$, $k_{2}$, $k_{3}$, $k_{4}$, and $k_{5}$ are correction factors to account for the radionuclide decay, the nuclide decay during counting, self-attenuation, pulses loss due to random summing, and the coincidence, respectively [15,16].

(2) $$\frac{u\left(A\right)}{A}=\sqrt{{\left(\frac{u\left(N\right)}{N}\right)}^{2}+{\left(\frac{u\left(P_{E}\right)}{P_{E}}\right)}^{2}+{\left(\frac{u\left(ℰ\right)}{ℰ}\right)}^{2}+{\left(\frac{u\left(T_{C}\right)}{T_{C}}\right)}^{2}+{\left(\frac{u\left(M\right)}{M}\right)}^{2}+{\left(\frac{u\left(k\right)}{k}\right)}^{2}}$$

where $\frac{u\left(N\right)}{N}$, $\frac{u\left(P_{E}\right)}{P_{E}}$, $\frac{u\left(ℰ\right)}{ℰ}$, $\frac{u\left(T_{C}\right)}{T_{C}}$, $\frac{u\left(M\right)}{M}$ and $\frac{u\left(k\right)}{k}$ are the relative uncertainties of the counting rate, gamma emission probability, photo peak efficiency, counting time, sample mass and correction factors, respectively. The standard uncertainty in the correction factors is determined as: ($\frac{u\left(k\right)}{k}=\sqrt{{\left(\frac{u\left(k_{1}\right)}{k_{1}}\right)}^{2}+{\left(\frac{u\left(k_{2}\right)}{k_{2}}\right)}^{2}+{\left(\frac{u\left(k_{3}\right)}{k_{3}}\right)}^{2}+{\left(\frac{u\left(k_{4}\right)}{k_{4}}\right)}^{2}+{\left(\frac{u\left(k_{5}\right)}{k_{5}}\right)}^{2}}$).

The standard uncertainties in activity measurements for ^40^K, ^210^Pb and ^137^Cs radionuclides were used for the determination of the expanded uncertainty. For ^226^Ra and ^232^Th which are determined from other radionuclides, the combined uncertainty was determined as the square root of the quadratic sum of the relative standard uncertainties of respective radionuclides as shown in Equations (3) and (4) [15,16].

(3) $$\frac{u\left(A_{\mathrm{Ra}-226}\right)}{A_{\mathrm{Ra}-226}}=\sqrt{{\left(\frac{u\left(A_{\mathrm{Pb}-214}\right)}{A_{\mathrm{Pb}-214}}\right)}^{2}+{\left(\frac{u(A_{\mathrm{Bi}-214})}{A_{\mathrm{Bi}-214}}\right)}^{2}}$$

(4) $$\frac{u\left(A_{\mathrm{Ra}-226}\right)}{A_{\mathrm{Ra}-226}}=\sqrt{{\left(\frac{u\left(A_{\mathrm{Pb}-212}\right)}{A_{\mathrm{Pb}-212}}\right)}^{2}+{\left(\frac{u(A_{\mathrm{Bi}-212})}{A_{\mathrm{Bi}-212}}\right)}^{2}+{\left(\frac{u(A_{\mathrm{Ac}-228})}{A_{\mathrm{Ac}-228}}\right)}^{2}}$$

Standard uncertainty in activity concentrations, as shown in Equation (2), was determined using software. The overall uncertainties in the measurement results were quoted as expanded uncertainty at 95% confidence level with coverage factor (k = 2) [15].

## 3. Results and Discussion

### 3.1. Radioactivity Contents in Marine Sediment

This study presents an effort to assess the magnitude of environmental and artificial radionuclides in marine environments. The specific activity (Bqkg^−1^) of natural radionuclides ^226^Ra, ^232^Th, ^40^K, and ^210^Pb in coastal marine sands and sediments in the Gulf of Oman are presented in Table 1.

**Figure 2:**
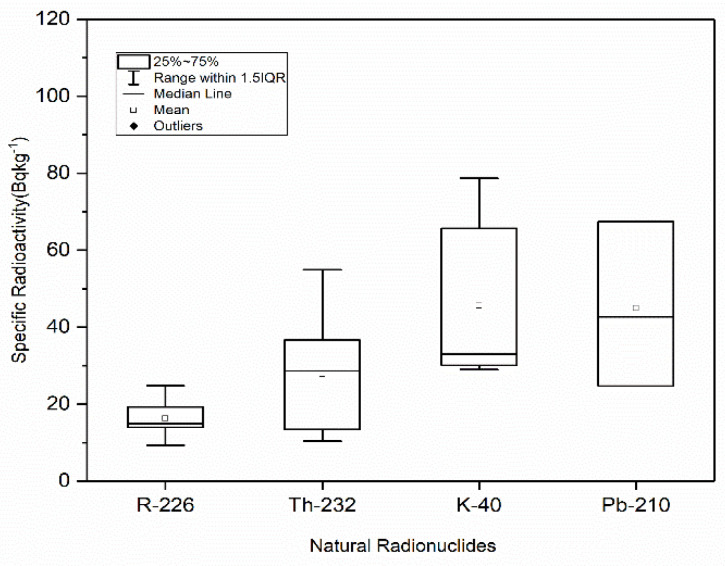
Boxplot of distributions of the radioactivity concentrations for ^226^Ra, ^232^Th, ^40^K, and ^210^Pb (natural radionuclides) in marine coastal sands.

**Figure 3:**
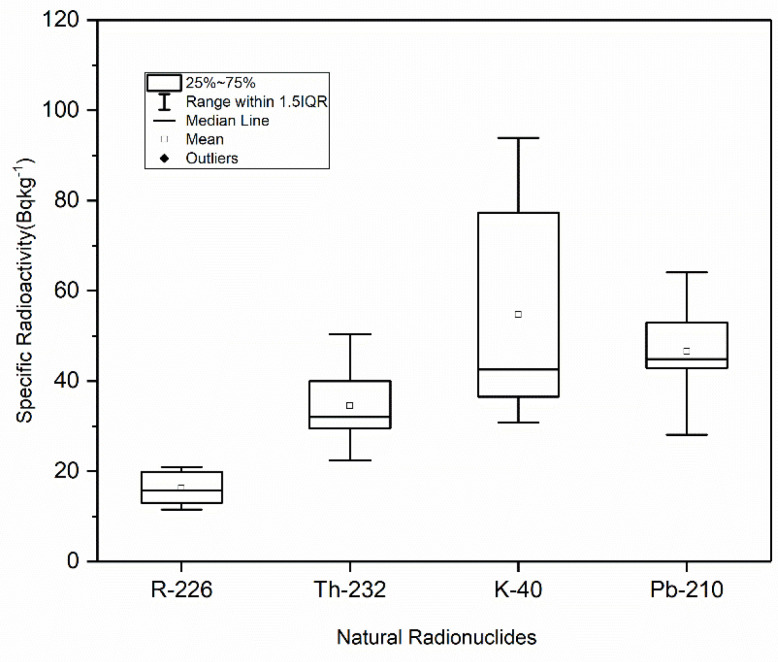
Boxplot illustrating the distributions of radioactivity concentrations for ^226^Ra, ^232^Th, ^40^K, and ^210^Pb natural radionuclides in marine sediments.

As shown, the radioactivity (Bqkg^−1^) of ^226^Ra, ^232^Th, ^40^K and ^210^Pb ranges were 9.324.8 (average: 16.3), 10.4–54.9 (average: 27.8), 29.0–78.7 (average: 45.6), and 24.7–67.4 (average: 44.9) Bqkg^−1^, respectively, in coastal sands, and from 11.0–21.0 (average: 16.2), 22.0–50.4 (average: 34.5), 30.8–93.4 (average: 54.7), and 28.1–65.1Bqkg^−1^ (average: 46.8), respectively, in marine sediments. Figure 2 and Figure 3 show the boxplot distribution of the radioactivity distribution of ^226^Ra, ^232^Th, ^40^K, and ^210^Pb radionuclides in sand and sediment, respectively. A large variability in activity concentrations is shown among radionuclides, reflecting the geological and morphological characteristics of the collected sediments, as well as their respective radionuclide contents. A high degree of variability in the measured radioactivity was shown in the studied samples, as these samples reflect the geological characteristics of their sites of origin. Usually, the radioactivity of ^238^U and ^232^Th is linked with heavy minerals, while that of ^40^K is associated with clay minerals.

The radioisotope of ^210^Pb revealed relatively high activity concentration (especially in the S05 and S07 sampling locations), considering a potential different origin than that of the local mineralogy, which could be due to submarine groundwater discharge sources in these areas.

In Table 2, a comparison is given of the average (range) radioactivity concentrations (Bqkg^−1^) obtained in this work versus in the literature. According to the IAEA, when the activity of the ^238^U or ^232^Th decay series is ≤1000 Bqkg^−1^and that of ^40^K is ≤10,000 Bqkg^−1^, the radioactive material may not be regarded as naturally occurring and is thus exempt from regulations [14]. The measured ^210^Pb in marine sediment originated from their parents ^222^Rn and ^226^Ra, which depend on several natural and environmental processes [17]. The radioactivity of ^210^Pb in the present work is comparable to values reported in the literature [9,10,11,12,13,17,18,19].

**Figure 4:**
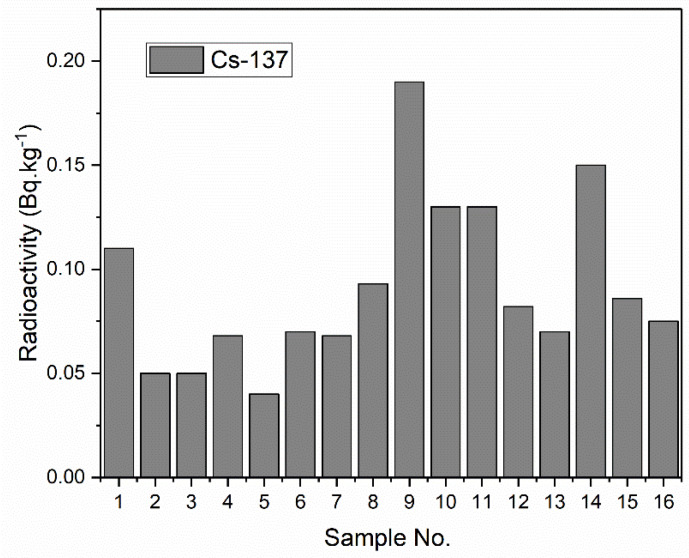
Bar charts of the radioactivity concentration of the artificial radionuclide, ^137^Cs.

Figure 4 shows a bar chart illustrating the distribution of activity concentrations of ^137^Cs among different samples. As shown, the radioactivity concentration of the artificial radionuclide ^137^Cs varied from 0.04–0.19 Bqkg^−1^ (average: 0.09). The ^137^Cs radioactivity levels in the current study were very low in most samples, suggestive of a low level of contamination. ^137^Cs in marine and other environments may have radiological impacts given its long half live, high yield, and high uptake and retention in biological systems. The results of our study were compared with the results of similar studies reported in different countries around the world (Table 2). As shown, the radioactivity levels in our study are comparable to those reported in Kuwait, Qatar, Saudi Arabia, and Greece, and can be explained by the fact that these studies were carried out in adjacent marine environments in which these radionuclides could be easily transported.

**Figure 5:**
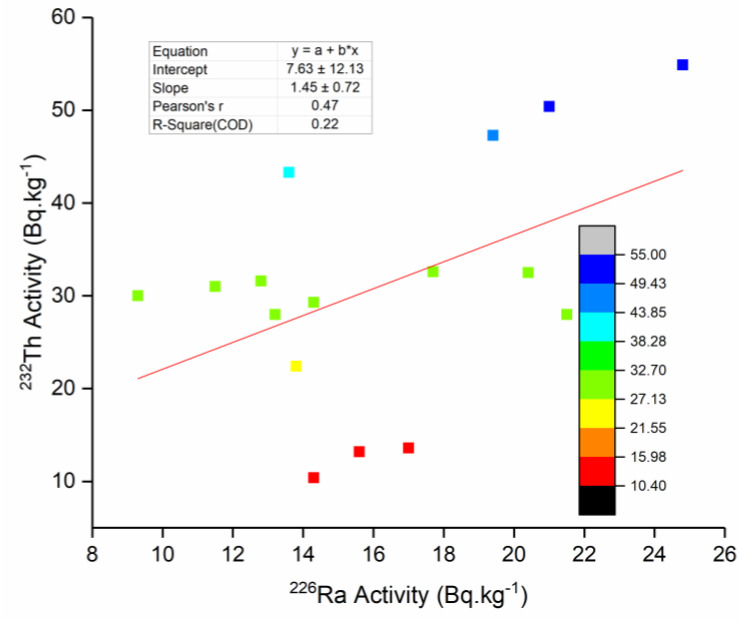
Correlation between the activity concentrations of ^226^Ra and ^232^Th (natural radionuclides); the colour codes correspond with ^232^Th activity.

**Figure 6:**
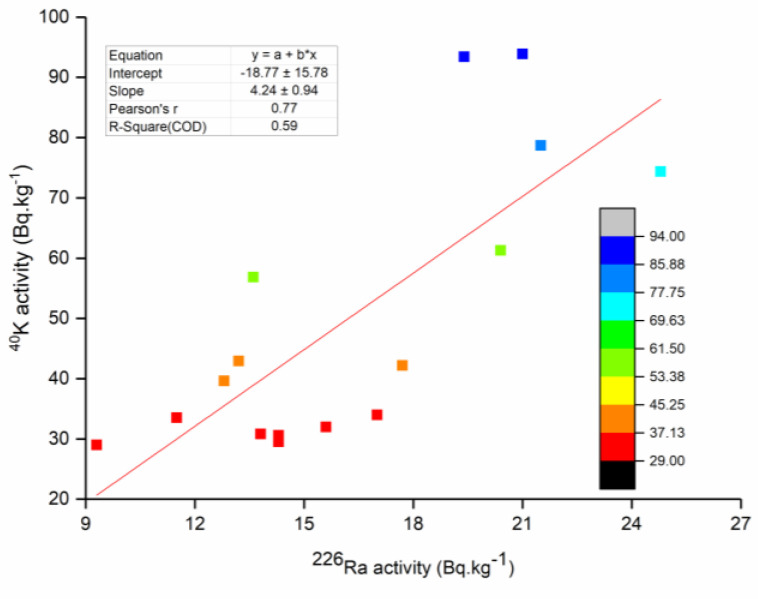
Correlation between the activity concentrations of ^226^Ra and ^40^K (natural radionuclides); the colour codes correspond with ^40^K activity.

Correlations between the activity concentrations of the radionuclides are presented in Table 3. The results are graphically depicted in Figure 5, showing the correlations between the activity concentrations of ^226^Ra and ^232^Th. Figure 6 shows the correlations between the activity concentrations of the naturally occurring radionuclides ^226^Ra and ^40^K are illustrated using corresponding colour codes.

The correlation between ^226^Ra and ^232^Th was not significant (*r* = 0.47, *p* > 0.05), whereas a highly significant correlation was observed between ^226^Ra and ^40^K (*r* = 0.77, *p* < 0.001) and between ^232^Th and ^40^K (*r* = 0.75, *p* < 0.001). The observed correlations could be attributed to the origin of these radionuclides.

### 3.2. Assessing Radiological Hazards

#### 3.2.1. Radium-Equivalent Activity

Radium-equivalent activity (Ra_eq_) is a single parameter that represents the collective risk of ^226^Ra, ^232^Th, and ^40^K radioactivity [21,22]. This parameter can be used to assess whether external doses to the public exceed the recommended annual dose limit of 1 mSv. The Ra_eq_ is determined using Equation (5):

(5) $${\mathrm{Ra}}_{\mathrm{eq}}=A_{\mathrm{Ra}}+1.43A_{\mathrm{th}}+0.077A_{K}$$

where A_Ra_, A_Th_, and A_k_ are the specific activities (Bq kg^−1^) of ^226^Ra, ^232^Th, and ^40^K, respectively, in the studied samples. Table 4 shows the radium equivalent activity (${\mathrm{Ra}}_{\mathrm{eq}}$), absorbed dose rates, effective rates, and external hazard index associated with the radioactivity in sand. As presented, the Ra_eq_ ranged from 31.5 to 109.0 Bqkg^−1^ (average: 59.6), with values < 370 Bqkg^−1^ representing the recommended limit for radiological risk control [2].

#### 3.2.2. External Hazard Index (H_ex_)

The external radiation exposure due to natural radioactivity is defined in terms of the external hazard index (H_ex_), calculated as follows [2,23]:

(6) $$H_{\mathrm{ex}}=\left(\frac{A_{\mathrm{Ra}}}{370}+\frac{A_{\mathrm{Th}}}{259}+\frac{A_{K}}{4810}\right)\leq 1$$

where A_Ra_, A_Th_, and A_k_ are the specific activities (Bq kg^−1^) of ^226^Ra, ^232^Th, and ^40^K, respectively in the studied samples. To comply with the requirements of the 1 mSv annual dose limit for the public, H_ex_ should be <1, as shown above [2]. As seen in Table 4, the H_ex_ values ranged from 0.09 to 0.30. These results ensure that the public’s exposure to the environmental radioactivity of ^226^Ra, ^232^Th, and ^40^K radionuclides in coastal sand remain within acceptable limits.

### 3.3. External Absorbed Dose Rates

The naturally occurring radioactivity in the environment is a major source of external exposure to the world’s population. The indoor (D_in_) and outdoor (D_out_) external gamma doses due to the presence of ^226^Ra, ^232^Th, and ^40^K in coastal sand 1 m above the ground surface can be calculated as follows [2,23]:

(7) $$D_{\mathrm{out}}\left({\mathrm{nGyh}}^{-1}\right)=0.427A_{\mathrm{Ra}}+0.662A_{\mathrm{Th}}+0.043A_{K}$$

(8) $$D_{\mathrm{in}}\left({\mathrm{nGyh}}^{-1}\right)=0.92A_{\mathrm{Ra}}+1.1A_{\mathrm{Th}}+0.081A_{K}$$

where A_Ra_, A_Th_, and A_k_ are the specific activities (Bq kg^−1^) of ^226^Ra, ^232^Th, and ^40^K, respectively, in the studied samples. As shown in Table 4, the $D_{\mathrm{in}}$ values (nGy.h^−1^) ranged from 27.0 to 89.2 (average: 49.26), whereas $D_{\mathrm{out}}$ values (nGy.h^−1^) ranged from 14.3 to 50.2 (average: 27.4). The current average dose figures fell below the global average value (55 nGy.h^−1^) for areas that were deemed to have normal levels of natural background radiation. Our results are lower than the doses reported by in Pakistan (87.47 nGy.h^−1^) [24]. According to UNSCEAR reports, a conversion coefficient, absorbed dose to effective dose received by adults of 0.7 Sv/Gy, and an outdoor occupancy factor of 0.2 were used [2]. Thus, the annual effective radioactivity dose in coastal sand can be estimated according to the following equations:

(9) $${\mathrm{AED}}_{\mathrm{out}}\left({\mathrm{Svy}}^{-1}\right)=D_{\mathrm{out}}\left({\mathrm{nGyh}}^{-1}\right)\times 8760{\mathrm{hy}}^{-1}\times 0.2\times 0.7{\mathrm{SvGy}}^{-1}\times 10^{-3}$$

(10) $${\mathrm{AED}}_{\mathrm{in}}\left({\mathrm{Svy}}^{-1}\right)=D_{\mathrm{in}}\left({\mathrm{nGyh}}^{-1}\right)\times 8760{\mathrm{hy}}^{-1}\times 0.8\times 0.7{\mathrm{SvGy}}^{-1}\times 10^{-3}$$

(11) $${\mathrm{AED}}_{\mathrm{total}}\left({\mathrm{Svy}}^{-1}\right)={\mathrm{AED}}_{\mathrm{out}}+{\mathrm{AED}}_{\mathrm{in}}$$

The total effective dose and ${\mathrm{AED}}_{\mathrm{Total}}$ (µSvy^−1^) ranged from 150.2 to 498.9 (average: 275.2) (Table 4). The global average annual effective dose from natural radionuclides (i.e., the sum of effective doses from both indoor and outdoor occupations) is 0.48 mSvy^−1^. The results for individual countries generally fall within the range of 0.3–0.6 mSv. The effective dose value obtained in this study is almost half of the value reported in Pakistan (0.92 mSvy^−1^) [24]. The effective dose is an important dosimetric quantity that allows different ionising radiation exposure categories to be compared and can be used to obtain broad estimates of radiation-attributed cancer incidents.

### 3.4. Excess Lifetime Cancer Risk (ELCR)

Low doses of ionising radiation, such as those encountered in response to natural radioactivity, are known to cause stochastic effects in the form of cancer. The probability with which these risks occur increases with increasing doses. The International Commission on Radiological Protection (ICRP) has estimated the number of fatalities per 1 Sv effective dose to be 0.05; this is known as the fatal risk factor. The ELCR can thus be determined using Equation (12) [2,23]:

(12) $$\mathrm{ELCR}={\mathrm{AED}}_{\mathrm{total}}\left({\mathrm{Svy}}^{-1}\right)\times \mathrm{LF}\times \mathrm{RF}({\mathrm{Sv}}^{-1})$$

where ${\mathrm{AED}}_{\mathrm{total}}\left({\mathrm{Svy}}^{-1}\right)$ is the annual effective dose calculated from indoor and outdoor exposure, LE is life expectancy (66 years), and RF is fatal risk factor per Sievert, which is 0.05 Sv^−1^, as per ICRP Report [6]. The average number of ECR per million population ranged from 58–203 (average: 111) due to radioactivity from sand (Table 4).

### 3.5. Annual Gonadal Dose Equivalent (AGDE)

The AGDE was computed from activity using Equation (13) [2,23]:

(13) $$\mathrm{AGDE}\;\left(\mathrm{mSv}.y^{-1}\right)=3.09A_{\mathrm{Ra}}+4.18A_{\mathrm{Th}}+0.314A_{K}$$

where A_Ra_, A_Th_, and A_k_ are the specific activities (Bq kg^−1^) of ^226^Ra, ^232^Th, and ^40^K, respectively, in the studied samples. The average AGD (µGy.y^−1^) ranged from 97.3 to 329.5 (average: 181.1) in coastal sand (Table 4). The global value is about 300 µGy.y^−1^ according to UNSCEAR reports [2].

### 3.6. Radiological Risk to Non-Human Biota

We have used the ERICA Tool software (Environmental Risk from Ionising Contaminants: Assessment and Management) to estimate the radiological risk parameters to non-human biota in marine environments [25]. ERICA Tool is a dosimetric model that enables calculations of internal and external absorbed dose rates to non-human biota covering a wide range of body masses and habitats for all radionuclides of interest. In addition, the software estimates the activity concentrations in biota; total absorbed dose rates, and risk quotients from the media (sediment) activity concentrations.

Table 5 shows the activity concentration in reference organisms in the marine environment determined using ERICA Tool. Total absorbed dose rate per organism as well as risk coefficients to non-human biota are presented in Table 6.

As shown in Table 5, the highest radioactivity was evident in phytoplankton, followed by benthic fish. The levels of ^210^Pb were significantly high in phytoplankton compared to those of the sediments indicating high ^210^Pb bioaccumulation in phytoplankton as suggested in the literature [26,27].

Table 6 presents the total absorbed dose rate to marine organisms and risk quotients due to radioactivity in marine sediments calculated using the ERICA Tool.

As shown, excluding phytoplankton, the estimated total dose rate per organism was below the background dose rates (Table 6). However, the total dose rate for phytoplankton exceeds the background dose rate by 48 %, which is due to the radioactivity bioaccumulation in phytoplankton. Thus, the total dose rate and risk quotients are comparable to those presented by Botwe et al. [28] in Ghana.

## 4. Conclusions

To recapitulate, radioactivity levels were determined for common natural and anthropogenic radionuclides in costal sand and marine sediments. The results show varying levels of natural radioactivity that were comparable to those reported in similar studies. A significant correlation was shown for ^232^Th and ^40^K, and for ^226^Ra and ^232^Th; these relationships could be attributed to the origin of these radionuclides. The radioactivity levels in sediments are a source of radiation exposure for marine organisms. Regular monitoring of radioactivity levels is vital for radiation risk confinement. The results provide important baseline data to which future radioactivity levels in marine environments can be compared. Considering the fact that oceans and seas form the ultimate sink of contaminants, including radioactivity, future research initiatives that study radioactivity levels in marine environments and assess associated radiological hazards to the population are of utmost importance in order to ensure protection of the marine environment. Such a project should also consider investigating radioactivity from artificial radionuclides.

## Figures and Tables

**Table 1:** Radioactivity concentrations (Bqkg^−1^) of ^226^Ra, ^232^Th, ^40^K, ^210^Pb, and ^137^Cs in coastal marine sands and sediments.

Sample Code	Weight (kg)	Activity Concentrations (Bqkg^−1^)				
		^226^Ra	^232^Th	^40^K	^210^Pb	^137^Cs
Beach Sand						
S01	1421	21.5 ± 1.4	28.0 ± 2.8	78.7 ± 4.8	**	0.11
S02	1371	24.8 ± 1.2	54.9 ± 3.1	74.4 ± 3.2	42.7 ± 19.4	0.05
S05	1592	14.3 ± 1.0	29.3 ± 2.2	29.5 ± 2.0	(125.5 ± 12.2)	0.04
S06	1568	14.3 ± 1.0	10.4 ± 1.1	30.6 ± 2.1	24.7 ± 11.4	0.07
S09	1501	13.6 ± 0.9	43.3 ± 3.3	56.9 ± 3.7	67.4 ± 13.5	0.19
S10	1414	9.3 ± 0.7	30.0 ± 2.6	29.0 ± 2.1	**	0.13
S13	1038	15.6 ± 1.0	13.2 ± 1.3	32.0 ± 2.1	**	0.07
S14	1376	17.0 ± 1.1	13.6 ± 1.3	34.0 ± 2.3	**	0.15
Average		16.30	27.84	45.64	44.9	0.10
Marine sediments						
S03	1363	21.0 ±1.0	50.4 ± 3.0	93.9 ± 3.8	44.9 ± 1.9	0.05
S04	1425	19.4 ± 1.4	47.3 ± 3.8	93.4 ± 5.7	**	0.07
S07	1532	12.8 ± 0.8	31.6 ± 1.2	39.6 ± 2.3	(158.9 ± 12.9)	0.07
S08	1750	11.5 ± 0.8	31.0 ± 2.4	33.5 ± 2.6	42.9 ± 12.5	0.09
S11	1496	13.8 ± 0.8	22.4 ± 2.2	30.8 ± 2.8	53.0 ± 11.4	0.13
S12	1421	13.2 ± 1.0	28.0 ± 1.8	42.9 ± 2.0	**	0.08
S15	1332	17.7 ± 1.4	32.6 ± 2.6	42.2 ± 3.9	65.1 ± 14.2	0.09
S16	1423	20.4 ± 1.2	32.5 ± 1.6	61.3 ± 2.7	28.1 ± 13.3	0.08
Average		16.2	34.5	54.7	46.8	0.08

The results in the brackets ( ) are outliers and are excluded from the average; ** indicate that the activity is less than the minimum detectable activity (MDA).

**Table 2:** Comparison of average (range) radioactivity concentrations (Bqkg^−1^) obtained in this work versus those in the literature.

Location	^226^Ra	^232^Th	^40^K	^210^Pb	^137^Cs	References
World	35	30	400	**	**	[2]
Qatar	4.2–19.5	1.0–6.0	11–188	**	0.18–0.66	[9]
Kuwait	17.3–20.5	15–16.4	353–445	23.6–44.3	1.0–3.1	[10]
Iran	11.8–22.7	10.7–25	223–535	**	0.14–2.8	[11]
Saudi Arabia	4.4–19.3	5.3–58.9	324.6–1133	**	0.6–8.7	[12]
Kuwait	18.6–21.4	14.0–17.1	351.2–404.0	**	1.5–2.9	[13]
Greece	18–86	20–31	368–610	47–105	0.7–3.8	[17]
China	13.7–52.	26.1–71.9	392–898	**	**	[19]
Egypt	38.51	**	33.35	659.18	**	[20]
Oman	16.2 (16.3)	34.5 (27.8)	54.7 (45.6)	46.8 (44.9)	0.1 (0.1)	This stud

** indicate that the activity is less than the minimum detectable activity (MDA).

**Table 3:** Correlation between activity concentrations of natural radionuclides.

Radionuclide	Statistics	^226^Ra	^232^Th	^40^K
^226^Ra	Correlation coefficient	1	0.47	0.77
	*p*-value	-	0.07	<0.001
^232^Th	Correlation coefficient	0.47	1	0.75
	*p*-value	0.07	-	<0.001
^40^K	Correlation coefficient	0.77	0.75	-
	*p*-value	<0.001	<0.001	<0.001

**Table 4:** Radium-equivalent activity, absorbed dose rates, effective rates, excessive cancer risk, annual gonadal dose and external hazard index (H_ex_) associated with the radioactivity in coastal sand.

Sample Code	R_aq_ (Bqkg^−1^)	Dose Rate (nGy.h^−1^)		$AED_{Total}$ (µSvy^−1^)	ELCR per 10^−6^	AGD µGy.y^−1^	H_ex_
		$D_{in}$	$D_{out}$				
S01	67.6 ± 5.7	56.9	31.1	317.2	126	208.2	0.18
S02	109.0 ± 4.6	89.2	50.2	498.9	203	329.5	0.30
S05	58.5 ± 3.1	47.7	26.8	267.1	108	175.9	0.16
S06	31.5 ± 2.6	27.0	14.3	150.2	58	97.3	0.09
S09	79.5 ± 5.0	64.7	36.9	362.6	149	240.9	0.22
S10	54.4 ± 3.4	43.9	25.1	246.0	101	163.2	0.15
S13	36.9 ± 2.7	31.4	16.8	174.8	68	113.4	0.10
S14	39.1 ± 2.9	33.3	17.7	185.2	72	120.1	0.11
Average	59.56	49.26	27.4	275.2	111	181.1	0.16

Raq is the radium equivalent activity, $D_{in}$ and $D_{out}$ are the indoor and outdoor air absorbed, respectively. $AED_{Total}$ is the total effective doses due to internal and external radiation exposure. ECR, excessive cancer risk. AGD (µGy.y^−1^), annual gonadal dose and Hex is the external hazard index.

**Table 5:** Activity concentration in organism [Bq kg^−1^ f.w.].

Isotope	Activity in Sediment (Bqkg^−1^ d.w.)	Activity Concentration in Organism (Bq kg^−1^ f.w.)					
		Benthic Fish	Macroalgae	Mollusc-Bivalve	Pelagic Fish	Phytoplankton	Zooplankton
Ra-226	16.2	0.43	0.27	0.20	0.43	3.48	0.25
Th-232	34.3	0.01	0.020	0.01	0.01	3.15	0.031
Pb-210	46.80	5.80	0.18	1.11	5.80	84.3	2.99
Cs-137	0.08	0.0006	0.0007	0.0004	0.0006	0.0001	0.0010

**Table 6:** Total dose rate per organism and risk coefficients due to radioactivity in marine sediments calculated using the ERICA Tool.

Organism	Background Dose Rates	Screening Value [µGy h^−1^]	Total Dose Rate per Organism [µGy h^−1^]	Risk Quotient
Benthic fish	0.58	10	0.067	0.007
Macroalgae	0.87	10	0.048	0.005
Mollusc-bivalve	2.0	10	0.036	0.004
Pelagic fish	0.42	10	0.059	0.006
Phytoplankton	0.38	10	0.564	0.056
Zooplankton	0.94	10	0.035	0.003

## Data Availability

The data presented in this study are available on request from the corresponding author. The data are not publicly available due to privacy reasons.
